# Clients’ Experiences and Satisfaction with an Integrated Intensive Outpatient Program for Substance Use Disorders

**DOI:** 10.62641/aep.v53i2.1835

**Published:** 2025-03-05

**Authors:** Kourosh Bador, Catrin Johansson, Ida Axelsson, Maja Nilsson, Nóra Kerekes

**Affiliations:** ^1^Centre for Holistic Psychiatry Research (CHoPy), 431 60 Mölndal, Sweden; ^2^AGERA KBT, 411 38 Gothenburg, Sweden; ^3^Department of Health Sciences, University West, 461 86 Trollhättan, Sweden

**Keywords:** addiction care, cognitive behavioral therapy (CBT), client satisfaction, integrated care, intensive outpatient program, substance use disorder (SUD)

## Abstract

**Background:**

Comorbidity between substance use disorders and other mental health conditions is common, yet existing treatments often fail to address its full spectrum. Opportunities for integrated treatment are limited, and the effects of such treatment remain relatively unexplored. This study explores the experiences of individuals with substance use disorders who successfully completed a four-month integrated intensive program at an outpatient addiction-care clinic in western Sweden.

**Method:**

An anonymous survey combining quantitative measures and qualitative open-ended questions was used to evaluate the experiences of 65 clients (out of 117) who completed the program between 2015 and 2021.

**Results:**

The findings revealed that most clients expressed high levels of satisfaction with the program. The mean scores for the questions ranged from 9.17 to 9.35, indicating a generally positive experience. The standard deviations were relatively low (1.17 to 1.34), suggesting consistency in responses. The median scores for all questions were 10, with ranges indicating that most participants rated their experiences at the highest level. The analysis identified three key categories of clients’ experiences: (1) strong relationships and a comprehensive treatment approach; (2) engaged, knowledgeable staff who lead with warmth; and (3) opportunities for self-development through novel experiences.

**Conclusion:**

Clients who successfully completed the four-month integrated intensive program reported high satisfaction levels, positive relationships with staff, and valuable self-development insights. However, the high dropout rate limited gaining an understanding of the barriers to program completion, highlighting the need for further research aimed at enhancing retention rates and developing more effective integrated treatment interventions for individuals with substance use disorders.

## Introduction

Substance use disorders (SUDs) are a significant global public health challenge. In 2019, more than 35 million people worldwide were reported to have drug use disorders, yet only one in seven individuals received treatment [1]. In Sweden, where this study was conducted, the Swedish Council for Information on Alcohol and Other Drugs (CAN) estimated that, in 2021, approximately 11% of the population aged 17–84 met the criteria for alcohol use disorder, and approximately 2% met the criteria for drug use disorder [2]. These figures show no significant change in the prevalence of SUD compared to data from 2017 [2].

SUDs frequently co-occur with other psychiatric conditions, with approximately 50% of individuals with alcohol and/or drug dependence experiencing comorbid psychiatric disorders [3]. Comorbidity presents unique challenges, as these individuals often require multifaceted interventions, including social, psychological, and pharmacological support [4]. Moreover, evidence indicates that individuals with mental health conditions face an elevated risk of developing an SUD [3]. These complexities highlight the importance of adopting patient-centered approaches that address the broader psychosocial and mental health needs of individuals with SUDs [5, 6].

Integrated care has emerged as a crucial framework for addressing these multifaceted needs. This involves coordinating multiple types of treatment—such as medical, psychological, and psychosocial interventions—to address both substance use disorders and co-occurring conditions in a cohesive manner [4]. The Swedish National Board of Health and Welfare recommends integrated programs that combine evidence-based approaches, such as medication, cognitive behavioral therapy (CBT), and psychosocial support for individuals with SUDs and concurrent psychiatric issues [7].

Two of the most widely used interventions in addiction care are the 12-step treatment program and CBT [7]. The 12-step treatment program, based on the approaches of Alcoholics Anonymous and Narcotics Anonymous, emphasizes abstinence, self-awareness, and peer support [8]. Participants are encouraged to reflect on their relationship with substance use, identify barriers to recovery, and actively seek help. CBT, a psychotherapeutic treatment proven effective for individuals with SUDs and comorbid psychiatric conditions, aims to help clients develop healthier cognitive and behavioral patterns to enhance their mental health and well-being [7, 9].

In addition to these established methods, complementary healthcare approaches, such as mindfulness and body-based interventions, are often incorporated into integrated care to address the symptoms of depression, anxiety, and stress that are frequently associated with SUD [10, 11]. Despite the recognized value of integrated care, current addiction treatment programs often prioritize medical and CBT interventions, with limited opportunities for patients to participate in comprehensive programs that combine psychological, psychosocial, and complementary treatments. This lack of treatment options limits the extent to which existing programs address the full spectrum of needs among individuals with SUD and co-occurring psychiatric conditions.

Only a limited number of studies have provided an evidence base for integrated addiction treatment programs [12], and a significant gap remains in the evaluation of client experiences within these types of integrated programs. Understanding these experiences is essential to enhancing the knowledge and skills of healthcare and social service professionals working with individuals with SUDs and concurrent psychiatric conditions.

This study aims to address this gap by describing the experiences of individuals with SUDs who participated in an integrated intensive outpatient addiction care program. Studying client experiences allows for a deeper understanding of how integrated treatment programs can be optimized to meet the needs of individuals with SUDs, ensuring that care is both accessible and effective in addressing their complex challenges.

## Methods

### Study Design and Procedure

To assess the quality of the integrated intensive outpatient care treatment, clients were asked to complete an anonymous evaluation survey after cessation of the treatment, which included both quantitative and qualitative questions.

### Context

A structured integrated intensive treatment program for adults with substance use disorders (SUDs) is offered at an outpatient addiction clinic in western Sweden. Grounded in the principles of CBT, the program spans four months and involves intensive treatment conducted five days per week. Clients engage in a variety of structured activities, including 51 hours of psychoeducation, 70 mindfulness sessions to cultivate a mindful presence, 70 hours of group sessions focused on emotional modulation, and 51 group interventions and affect regulation. In addition, the program incorporates 17 individual psychotherapeutic sessions and 17 shared activity sessions to develop social skills, practice exposure, and conduct behavioral experiments. Complementary methods, such as 17 standardized acupuncture sessions for sleep, anxiety, and pain relief, as well as 17 yoga sessions aimed at enhancing body awareness, balance, self-regulation, and conscious breathing, are also included. Additional practices, such as tai chi, further enrich the program’s holistic approach. Delivered by trained clinical professionals, the treatment aims to support individuals in navigating life challenges, addressing the root causes of their difficulties, and experiencing more meaningful and fulfilling lives.

### Data Collection

The survey consisted of six questions, the first three of which were answered on a Likert scale [13], where clients were asked to rate their experience on a numbered scale from 0 (not satisfied at all) to 10 (very satisfied).

To ensure content validity, the questions were formed to align with the clinic’s objectives, aiming to accurately capture the participants’ experiences and perceived learning. Construct validity was enhanced by using targeted questions, such as the first question “How did you experience your time at the clinic?” and the second question “How did you experience the program?” These questions were designed to assess the participants’ overall satisfaction and level of engagement with the program. The third question aimed to gauge the participants’ perceptions of how effectively they absorbed the information, directly reflecting the educational goals of the program.

Although a formal reliability test was not conducted due to the study’s exploratory nature, the questionnaire was piloted during the first year (2014) of the program, involving the first clients of the clinic, to improve clarity and ensure consistent interpretation across the respondents.

Data accuracy was maintained by inviting the clients to complete the questionnaire on paper, alone and anonymously, at the end of the program prior to leaving the clinic. This approach was intended to minimize social desirability bias and promote honest feedback. The responses were then continuously entered into Microsoft Excel (365 version, Microsoft Corp., Redmond, WA, USA), with raw data archived in accordance with the Swedish Patient Data Act, to ensure confidentiality and data integrity [14].

The remaining three questions were open-ended, allowing clients to write about and expand on their experiences with the treatment. There was also an opportunity to provide additional valuable information and comments about their experiences in the final question.

### Data Analysis

#### Statistical Analyses

The clients responded to first three questions using a 0–10 ordinal scale (0 = not satisfied at all, 10 = very satisfied). Their responses were analyzed and reported using descriptive statistics. IBM SPSS Statistics (version 29, IBM Corp., Armonk, NY, USA) was utilized to calculate the mean (M), standard deviation (SD), median (Md), and interquartile range (IQR, Q1–Q3).

#### Qualitative Content Analysis

The qualitative analysis of the free-text responses from 65 clients was guided by Graneheim and Lundman’s (2004) [15] approach. The process began with the identification of “meaning units”, defined as sentences or paragraphs encapsulating the core content of the text. These meaning units were then condensed to reduce the volume of text while retaining the essential information. Next, the condensed meaning units were systematically coded to clarify their content and establish logical themes. Codes with similar meanings were grouped collaboratively to form three overarching categories, as presented in Table 1.

**Table 1.** **Overview of the analysis process**.

Meaning unit	Condensed meaning unit	Code	Category
This has been a highly educational experience, providing a wealth of tools not only for sobriety but for life in general. We have formed a wonderful group, and although there may be individuals with whom I don’t feel secure at times, that has not been the case here.	Enriching and supportive, both for sobriety and life. This has been a wonderful group in which I have felt safe.	Satisfied with the experience of the treatment program and the relationships that have been formed.	Strong relationships and a comprehensive treatment program.
The healthcare staff have been crucial for me, looking straight into my addiction persona and helping me unveil everything, without keeping any secrets.	The approach of the healthcare staff has been important in understanding the entirety of my issues.	Appreciate the healthcare staff.	Dedicated, knowledgeable personnel who lead with warmth.
The healthcare staff have helped and supported me, guiding me to think differently.	They have assisted me to think differently.	New ways of thinking.	Personal development through new experiences.

## Results

### Descriptive Statistics

Between 2015 and 2021, a total of 117 individuals (45 women and 72 men, mean age 41.27, SD = 11.9) started treatment. Of these, 52 did not complete the four-month program, resulting in a final study population of 65 adults who completed the integrated intensive outpatient treatment and anonymous program evaluation survey. This group included 26 women (mean age 41.7, SD = 12.4) and 39 men (mean age 42.6, SD = 13.0). Among these participants, 71.8% of the men and 65.4% of the women were single. Additionally, 41.0% of the men and 30.8% of the women had no children.

Clients who completed the four-month intensive integrated treatment program (N = 65) were asked to reflect on their experiences at the clinic, their engagement with the treatment program, and their ability to comprehend and internalize the information provided. These findings are summarized in Table 2. The results demonstrated high levels of satisfaction with the integrated intensive outpatient treatment.

**Table 2.** **Clients’ satisfaction with a four-month integrated intensive outpatient treatment program**.

Questions	M (SD)	Md (IQR)
How did you experience your time at the clinic?	9.35 (1.20)	10 (9–10)
How did you experience the treatment program?	9.17 (1.34)	10 (8–10)
Do you think you were able to take in what was conveyed?	9.27 (1.17)	10 (9–10)

M, mean; Md, median; SD, standard deviation; and interquartile range (IQR, Q1–Q3) scores on a 0–10 scale (0 = not satisfied at all, 10 = very satisfied).

All 65 participants (100%) responded to the first question, “How did you experience your time at the clinic?” The mean score for their experiences at the clinic was 9.35, with the majority (70.8%) rating their satisfaction at the highest level (10). The lowest score for this question was 5.

Similarly, participants evaluated their experiences with the treatment program (question 2) positively, with a mean score of 9.17. All clients responded to this question, and 65% rated their satisfaction at the highest level (10). The minimum score for this response was also 5.

The third question assessed how well the clients felt they had absorbed the information provided during the program. A total of 63 participants (97%) responded, yielding a mean score of 9.27. Of these, 61% rated their satisfaction at the highest level (10), indicating that the information was communicated in a clear and comprehensible manner. The minimum score reported for this response was 5.

### Qualitative Content Analysis

The qualitative analysis of the study identified three overarching categories: strong relationships and a comprehensive treatment program, engaged and knowledgeable staff who lead with warmth and security, and self-development through new experiences. These themes reflect the clients’ overall perceptions of and experiences with the program.

### Strong Relationships and a Comprehensive Treatment Program

The participants expressed positive perceptions of the program’s structure and design, emphasizing its individualized approach and the inclusion of diverse elements, such as group sessions, lectures, individual therapy, mindfulness exercises, and physical activities, such as yoga and tai chi. The variety and integration of these components were highly valued.

Many participants highlighted the sense of community that developed within their treatment groups, describing the bonds formed as a source of joy and support. Some mentioned that they had developed lifelong friendships through the program. Additionally, they expressed a desire for more shared activities, such as tai chi, both during the program and in their free time.

The program’s impact on the participants’ lives was profound, with most expressing deep gratitude for the opportunity to take part. Some indicated that they would eagerly recommend the program to others and even expressed a willingness to rejoin if possible. The program was viewed as a significant contributor to enhancing their overall quality of life.

### Engaged, Knowledgeable Staff Who Lead with Warmth and Security

A common theme among the participants was the sense of security fostered by the staff’s professionalism and dedication. Key characteristics of the staff, including their openness, warmth, and expertise, were frequently mentioned as integral to the participants’ positive experiences.

The participants particularly valued the staff’s ability to create a secure and stable environment, especially in group settings where some clients might typically feel vulnerable. The staff’s commitment to understanding and supporting clients made the participants feel heard, seen, and respected. This trust-building approach was pivotal in strengthening the therapeutic relationship and the overall experience of the program.

### Self-Development through New Experiences

The clients reported significant personal growth and development as a result of the program. They described gaining new experiences, an enhanced self-image, and greater insight into their issues and illness. The program was seen as a gateway to adopting healthier lifestyles and a source of hope for a better future.

Self-reflection emerged as a particularly valued aspect of the program, allowing clients to reevaluate their situations and make meaningful changes. Many clients expressed feeling as though the program had given them a “fresh start”, enabling them to approach life with a renewed perspective and optimism.

## Discussion

The findings of this study reveal that clients who underwent integrated intensive outpatient treatment expressed high satisfaction with the program, its staff, and the transformative processes they experienced. Clients valued the diverse elements of the program, which included psychoeducation, group and individual therapy, mindfulness, yoga, tai chi, and acupuncture. The integration of complementary methods alongside conventional treatments reflects a growing international trend [16]. However, in Sweden, the frequency of their use in addiction care remains unclear. While complementary methods are generally associated with minimal side effects [17, 18], concerns have been raised about their potential to overshadow primary care [16]. Swedish patient legislation [19] regulates the use of such methods to ensure that they enhance rather than detract from conventional treatments.

The results highlighted the program’s ability to improve participants’ self-image and quality of life, consistent with Körkel’s [20] assertion that individuals with SUDs often struggle with motivation due to poor self-perception. The participants described the group sessions as a source of connection and support that fostered a sense of belonging and participation. This aligns with the findings from the Substance Abuse and Mental Health Services Administration [21], which underscores the importance of group dynamics in addiction treatment. Group interactions provided a space for sharing experiences and breaking the isolation often associated with substance use disorders. However, some participants noted the potential for discomfort in group settings, particularly fears of judgment and confrontation, which could diminish the therapeutic benefits if not addressed [21].

Staff played a pivotal role in the positive client experiences reported. The warmth, openness, and expertise of the staff were frequently mentioned as contributing to a sense of security and stability. This aligns with the concept of person-centered care [22, 23], which emphasizes addressing individual strengths, goals, and desires. The strong therapeutic alliance between staff and clients was crucial for motivating the participants to complete the program. Previous research [24] highlights the importance of mutual vulnerability in therapeutic relationships, such that clients rely on staff for support, and staff take on caregiving roles, thus creating a shared path toward recovery. This approach differentiates integrated programs from the 12-step model, in which peer accountability and shared experience are central, and from standalone CBT, which may lack the relational depth emphasized in integrated care.

The findings also underscore the need to address co-occurring psychiatric conditions in addiction treatment. Traditional approaches often focus narrowly on the primary diagnosis, overlooking secondary mental health challenges [22]. Integrated programs, by incorporating mental health support alongside addiction treatment, offer a more comprehensive solution [4]. However, the limited availability of such programs in Sweden constrains their reach and potential impact.

Compared to traditional treatments, such as 12-step treatment programs or CBT alone, the integrated approach offers a more comprehensive and adaptable therapeutic framework. The 12-step model focuses on abstinence and peer support but may lack the individualized and multifaceted interventions that integrated programs provide. Similarly, while CBT emphasizes cognitive and behavioral modifications, it may not include the holistic physical and emotional components offered by integrated programs, such as yoga, mindfulness, and acupuncture. These additional elements address the diverse and complex needs of individuals with SUDs and comorbid mental health conditions, potentially fostering more sustainable recovery pathways.

Integrated treatment programs should prioritize combining evidence-based practices with holistic approaches, such as group therapy, psychoeducation, and mindfulness, and physical activities, such as yoga and tai chi. These components contribute to the development of strong interpersonal relationships, emotional growth, and improved self-image, which are essential for long-term recovery.

The pivotal role of engaged, knowledgeable, and warm staff highlights the need for robust training in person-centered care and trauma-informed practices. Staff should be equipped to build trust and foster a secure environment, particularly for clients who may feel vulnerable or uncertain in group settings.

To address the high dropout rates, targeted interventions should be developed to support individuals at risk of disengagement. This could include early identification of challenges, personalized follow-ups, and flexible entry points and tailored support for clients with complex needs.

Further studies should explore the long-term impact of integrated intensive programs on clients’ well-being and recovery. Research should also evaluate the scalability of such programs and their adaptability to different healthcare settings to ensure broader implementation.

By adopting these recommendations, integrated intensive outpatient programs can continue to evolve and play a critical role in addressing the complex needs of individuals with SUDs and comorbid mental health conditions, ultimately promoting recovery and enhancing quality of life.

Although challenges related to accessibility and implementation remain, the integrated model holds significant promise for improving addiction care by merging evidence-based treatments with complementary approaches. Research indicates that patients with SUDs and coexisting mental health conditions who fail to complete treatment or perceive its quality as poor are more likely to experience suboptimal outcomes [25]. This highlights the critical importance of client satisfaction, as those who complete treatment and report high satisfaction with the program are more likely to achieve positive outcomes, enhanced well-being, and improved recovery through integrated treatment interventions.

### Strengths and Limitations

This study has several limitations and strengths that should be considered when interpreting the findings.

A primary limitation is the modest size of the study population resulting from a high dropout rate, which impacts the generalizability of the results. High dropout rates are common in clinical research involving patients in SUD-specific programs or interventions, e.g., [26, 27]. However, a notable strength of the study lies in the substantial number of responses collected from open-ended questions, which provided valuable insights. That said, these responses may lack the depth and richness typically obtained through individual interviews, which represents a limitation.

A qualitative analysis inherently involves the interpretation of the narrative meaning [28]. Thus, the findings should be understood within their specific context—in this case, an outpatient clinic. Whether the results are transferable to other contexts, as outlined by Graneheim and Lundman [15], is ultimately a judgment for the reader.

Another limitation is the anonymity of the feedback collected at the conclusion of the four-month program. While anonymity likely encouraged honest and unbiased responses—since the clients were not expected to return to the clinic after completing their four-month treatment—it restricted the analysis, preventing the exploration of variations in feedback by demographic factors, such as gender.

Additionally, feedback was obtained only from clients who completed the program, excluding those who dropped out. The absence of input from individuals who did not finish the program limits gaining an understanding of their experiences and the barriers they encountered. Due to varying dropout timings and the lack of post-dropout contact with the clinic, gathering feedback from this group was not feasible, thus leaving a gap in the understanding of why some clients were unable to complete the treatment.

A unique strength of this study is that it is the first to explore clients’ perspectives on a CBT-based integrated intensive addiction treatment program in Sweden. The outpatient clinic under study is one of few—possibly the only—facilities offering this specific type of addiction treatment in the country. This distinction enhances the study’s relevance but also limits the sample size, as no other comparable clinics were available from which to recruit additional participants.

## Conclusion

This study underscores the substantial benefits of integrated intensive outpatient programs for individuals with SUDs. The findings demonstrate high levels of client satisfaction, highlighting three key factors: the importance of strong relationships and a well-structured treatment program, the pivotal role of engaged and knowledgeable staff, and the value of self-development through new experiences. While the results are encouraging, the study emphasizes the need for further research to deepen understanding and build competence in delivering integrated treatment interventions for individuals with SUDs and comorbid mental health conditions. This continued exploration is crucial for enhancing the effectiveness and accessibility of comprehensive care for this population.

## Availability of Data and Materials

The data supporting this study are not publicly available due to restrictions imposed by the Swedish Ethical Review Authority. However, the data may be accessed upon reasonable request to the corresponding author, KB.
